# Expression of thymidine phosphorylase in peripheral blood cells of breast cancer patients is not increased by paclitaxel

**DOI:** 10.1186/1472-6904-7-7

**Published:** 2007-07-18

**Authors:** Rupert Bartsch, Guenther G Steger, Birgit Forstner, Catharina Wenzel, Ursula Pluschnig, Blanka Rizovski, Gabriela Altorjai, Christoph C Zielinski, Robert M Mader

**Affiliations:** 1 First Department of Medicine and Cancer Centre, Clinical Division of Oncology, Medical University of Vienna, Vienna, Austria

## Abstract

**Background:**

A synergistic cytotoxic effect has been hypothesized for taxanes and capecitabine, a prodrug of 5-fluorouracil. Based on preclinical studies, this synergism has been attributed to an up-regulation of the enzyme thymidine phosphorylase (TP). Beside tumour tissue, TP is highly expressed in white blood cells, possibly causing increased hematotoxicity, when taxanes are combined with capecitabine. So far, this hypothesis has not been investigated in humans.

**Methods:**

A total of 128 consecutive blood samples were collected from eight patients with advanced breast cancer receiving paclitaxel weekly at a dose of 80 mg/m^2^. To assess the expression of TP in blood cells, samples were collected prior to first therapy, at the end of infusion, and up to 15 days thereafter. This procedure was repeated during the sixth application of paclitaxel. After isolation of the peripheral mononuclear blood cells, the expression of TP was assessed by ELISA. In parallel, paclitaxel level in plasma was evaluated at three selected time points as pharmacokinetic control parameter.

**Results:**

Paclitaxel concentrations at the end of infusion did not change significantly from week 1 to week 6. The expression of TP in peripheral mononuclear blood cells decreased significantly after infusion below pretherapeutic values (p = 0.023; n = 8). After the nadir on day 3, the expression of TP increased moderately returning to baseline levels within one week. The overall picture in week 6 was similar to week 1. Using a trend analysis, neither a short-term nor a long-term induction of TP was observed.

**Conclusion:**

TP in peripheral mononuclear blood cells was hardly regulated under therapy with paclitaxel. Therefore, no increased haematotoxicity due to TP upregulation is expected from the combination of taxanes and capecitabine.

## Background

In advanced breast cancer, combinations of cytotoxic agents with synergistic antitumour effect deserve particular attention. Different groups reported increased response rates and prolonged time to disease progression in combination therapy as compared to single-agent treatment. Yet, combining cytotoxic drugs is usually associated with increased toxicity. A synergistic effect has been hypothesized for paclitaxel and capecitabine, a prodrug of 5-fluorouracil. Taxanes are a group of compounds that interact with the β subunit of tubulin and induce tubulin polymerization thus interfering with the normal balance between polymerization and its contrary, depolymerisation. This mechanism eventually leads to the arrest in the G2/M phase of the cell cycle with subsequent cell death [1].

The parent drug capecitabine, on the other hand, has no intrinsic cytotoxic effect, but is activated via a cascade of three enzymes to yield the well-known anticancer agent 5-fluorouracil. The activation is completed by the enzyme thymidine phosphorylase (TP) converting 5'-deoxy-5-fluorouridine to 5-fluorouracil, preferentially within the tumour cell [2]. Beside a known elevated expression of thymidine phosphorylase within tumour cells and white blood cells [3], paclitaxel and other agents have the ability to induce this enzyme in the mouse model [4]. The very pronounced up-regulation in tumours due to paclitaxel should eventually lead to the synergistic effect of the combination paclitaxel and capecitabine [5,6]. If the same upregulation however holds true also for TP in white blood cells, the assumed synergistic activity would increase haematologic side effects of taxanes. Therefore several questions remain, which need to be answered in order to fully exploit the potential benefits of combining paclitaxel and capecitabine in therapeutic protocols. In particular, the scheduling of those substances appears important for a synergistic effect [7]. Clinical studies relying on the up-regulation of thymidine phosphorylase have already been published [6,8-10]. Yet, in contrast to data from tumour xenograft models, corresponding data from human tumour tissue is lacking. Furthermore, it must be asked whether a regulation of TP in white blood cells occurs. We have no knowledge concerning kinetics of a possible TP induction in human white blood cells (e.g. start of thymidine phosphorylase up-regulation, maximum expression level after single dose, decline of thymidine phosphorylase expression after a single dose of the modulating drug, maximum expression level reached under repeated administration).

There is a vast clinical experience with paclitaxel administered as a single agent in patients suffering from advanced breast cancer. Initially administered every three weeks at a dose of 175 mg/m^2^, dose density concepts have helped to establish a weekly schedule with relatively mild toxicities at 80 – 100 mg paclitaxel/m^2 ^[11,12] and in the elderly [13]. This concept has also been extended to combination trials of capecitabine with a variety of anticancer agents such as docetaxel, platinum drugs, anthracyclines, 5-fluorouracil, and others [14].

From previous studies, it is known that capecitabine distributes rapidly into malignant lesions with a mean delay of 45 minutes when compared with plasma kinetics [15]. As consequence, the putative up-regulation of thymidine phosphorylase expression by paclitaxel would translate into modulatory benefit with negligible time delay in humans. It is therefore reasonable to assume that once the induction of TP is established in humans, a more rational approach might further exploit the synergism of paclitaxel with capecitabine. On the other hand, optimal scheduling to reduce haematotoxicity caused by a hypothetical TP induction in white blood cells might also be possible.

Given the above described facts, we initiated this trial to answer questions concerning the kinetics of TP induction in human peripheral mononuclear cells by paclitaxel. In parallel to serial assessment of thymidine phosphorylase expression, three characteristic data points of paclitaxel pharmacokinetics were used as a pharmacokinetic quality control parameter.

## Methods

All specimen and data were collected at the First Department of Medicine and Cancer Centre, Clinical Division of Oncology, at the Medical University of Vienna, Vienna, Austria. The trial was performed in accordance with the ethical regulations of the Medical University of Vienna and has been approved by the appropriate ethics committee prior to its initiation.

### Patients

A total of eight patients with histological confirmed advanced or metastatic breast cancer were included from February 2005 until September 2005; all eight are evaluable for toxicity and response. Criteria for inclusion were as follows: histological confirmed metastatic or locally advanced inoperable breast cancer without prior taxane (paclitaxel, docetaxel) treatment (adjuvant or palliative), Karnofsky Performance Index > 80, adequate bone marrow function (WBC > 3.5 G/I, PLT > 100 G/l), adequate liver- and kidney function parameters (creatinine < 1.5 mg%, bilirubin < 2.0 mg%, ASAT < 2.5 × normal, PTT > 60%), serum calcium within normal ranges, and written informed consent. Patients under the age of 18 years, with prior chemotherapy (adjuvant or palliative) with taxanes, second neoplasias except cured basalioma of the skin or cervical cancer, treatment with any investigational drug during the last four weeks prior to recruitment to this study, serious illnesses (e.g. cardiac illness, inflammatory bowel disease, diabetes without sufficient treatment, seizures,...), pregnancy, inadequate contraception, and Iactation were excluded from the study.

### Treatment plan and patients evaluation

80 mg Paclitaxel/m^2 ^(Taxol^®^; Bristol-Myers Squibb, Princeton, NJ, USA) were administered intravenously by a 1- hour infusion. Premedication consisted of 8 mg dexamethasone i.v., 50 mg diphenhydramine i.v., and 50 mg ranitidine i.v. 30 minutes prior to paclitaxel administration. Serotonin receptor antagonists were administered according to clinical routine protocols. Paclitaxel was administered weekly for six weeks every eight weeks for a minimum of two cycles before restaging. Tumour response was assessed every 2 cycles using UICC criteria.

### Experimental design

To assess the expression of thymidine phosphorylase in blood cells, 8 ml blood were collected prior to therapy (day 1), at the end of the i.v. infusion, 24 h, 48 h, and 72 h later, as well as at days 8, 11, and 15. This procedure was repeated during the sixth application of paclitaxel (week 6).

To evaluate paclitaxel concentrations, samples of 3 ml blood were drawn prior to therapy, at the end of the i.v. infusion, 20 h, and 44 h after drug administration into EDTA containing vials. After collection, blood was centrifuged immediately for 5 min at 3000 RPM. The plasma was separated from blood cells and stored at -20°C until analysis.

Peripheral mononuclear blood cells were isolated by ficoll gradient centrifugation (Cell Preparation Tube System; Becton-Dickinson, Franklin Lakes, NJ, USA). Sodium citrate vials were used as blood containers. Recovered cells were washed twice in phosphate buffered saline, and lysed by sonification. As a reference, the content of total protein is determined using a colorimetric assay (BCA Protein Assay Kit; Pierce, Rockford, IL, USA). The expression of thymidine phosphorylase was assessed in duplicates using an ELISA according to the manufacturer's instructions (Thymidine Phosphorylase ELISA; Roche, Mannheim, Germany).

Paclitaxel was evaluated using an HPLC method developed in our laboratory [16]. Briefly, frozen samples were thawed under light protection, vortex-mixed, and centrifuged at 20,000 × g. 100 μl from the clear supernatant were directly injected onto the HPLC system and loaded onto a C4-ADS clean-up column with a constant flow of methanol – water. After column-switching 10 min later, paclitaxel was transferred in backflush mode onto the analytical column delivering ammonium acetate buffer – acetonitrile as a solvent. The analyte was evaluated by monitoring the UV-absorbance at a wavelength of 229 nm.

### Statistical analysis

The expression of thymidine phosphorylase was calculated as ng thymidine phosphorylase/mg protein of the cell lysate. Results are provided as mean +/- standard deviation, with outliers indicated. The kinetics of thymidine phosphorylase expression were monitored in paclitaxel naive patients and compared with the observed concentrations after six administrations of paclitaxel (paired Wilcoxon test). Paclitaxel concentrations monitored at the end of the infusion, one and two days later were compared with previously evaluated pharmacokinetic data to exclude outlying patients. In order to statistically describe significant courses in thymidine phosphorylase expression vs. time, a trend analysis was performed to test for slope deviations significantly different from zero. Statistics were calculated using GraphPad PRISM version 4.00 software (GraphPad Software, San Diego, CA, USA).

## Results

### Patient characteristics

Eight paclitaxel naive patients with a median age of 65.5 years (range: 53 – 73 years) suffering from advanced breast cancer were included in this evaluation. Weekly paclitaxel was used as first line therapy in 4 patients (50%), second line in 2 patients (25%), and beyond second line in 2 patients (25%). Table 1 lists the characteristics of the eight patients included.

**Table 1:** Patient characteristics

Characteristics	Patients
Entered	8
Karnofsky performance score	90–100%
Age (years)	
Median (range)	65.5 years (range 53 – 73 y)
Stage (primary diagnosis)	
I	-
II	6 (75%)
III	-
IV	1 (12.5%)
Not available	1 (12.5%)
Ductal/Lobular	7/0
Not available	1 (12.5%)
Grading	
1	-
2	2 (25%)
3	5 (62.5%)
Not available	1 (12.5%)
Estrogens receptor/progesteron receptor positive	7/6
Her2 Status positive/negative (IHC/FISH*)	0/8
Adjuvant chemotherapy	3 (37.5%)
Adjuvant endocrine therapy	3 (37.5%)
Palliative endocrine therapy	4 (50%)
Prior anthracycline exposure	5 (62.5%)
Prior capecitabine exposure	2 (25%)
Time to recurrence	
Median (range)	87 months (13 – 148 m)
Treatment line	
First line	4 (50%)
Second line	2 (25%)
>/= Third line	2 (25%)
Metastatic sites	
Median (range)	2 (range 1 – 4)
bones/soft tissue only	3 (37.5%)
visceral only	-
both	5 (62.5%)
Localisation	
Lung	1
Liver	3
Bones	5
Lymph nodes	6
Soft tissue	2
Brain	1
Skin	1
More than one metastatic site	7 (87.5%)

* IHC: immunohistochemistry, Herceptest^® ^(Dako A/S, Glostrup, Denmark); FISH: dual colour fluorescent in situ hybridization (PathVision^® ^HER2 DNA probe kit, Vysis, Downers Grove, IL, USA)

### Paclitaxel

The concentrations of paclitaxel at the end of the i.v. infusion did not change significantly from week 1 to week 6 indicating stable pharmacokinetics in the study population (c_max_: 3480 ± 1020 nmol/L vs. 3040 ± 1510 nmol/L). A similar observation was made when analyzing samples 24 h (plasma concentration: 33.1 ± 12.0 nmol/L vs. 28.9 ± 23.4 nmol/L) and 48 h later (plasma concentration: 8.6 ± 7.6 nmol/L vs. 10.9 ± 7.0 nmol/L).

### Thymidine phosphorylase

**Figure 1:**
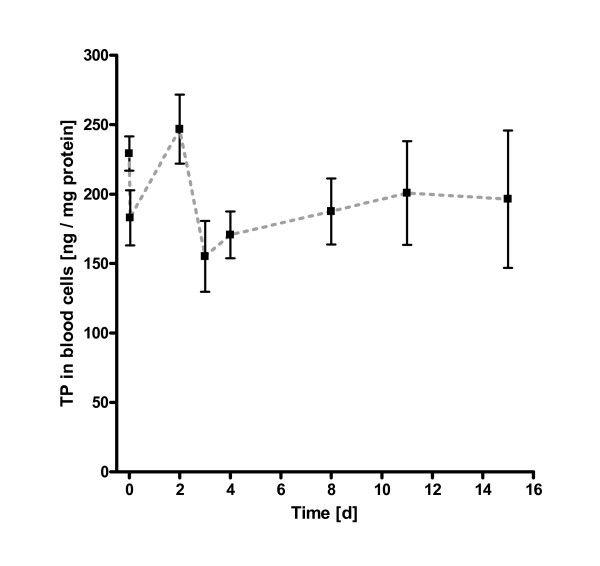
Thymidine phophorylase kinetics in peripheral mononuclear blood cells (n = 8); week 1. Arithmetic means ± standard error of mean (SEM).

**Figure 2:**
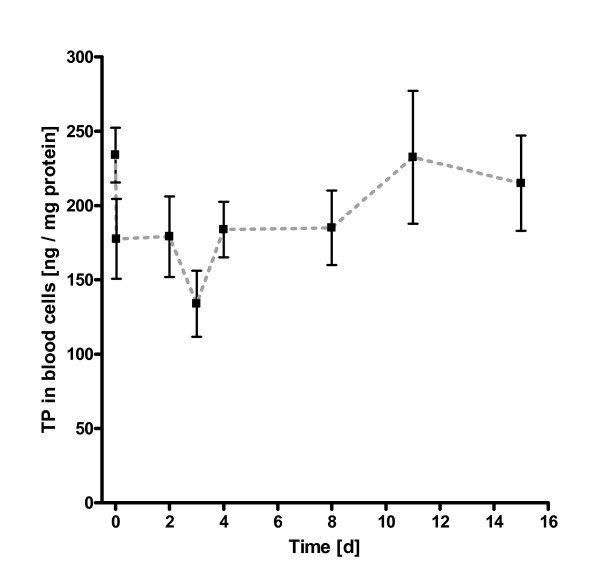
Thymidine phophorylase kinetics in peripheral mononuclear blood cells (n = 8); week 6. Arithmetic means ± standard error of mean (SEM).

In eight paclitaxel naive patients, the expression of thymidine phosphorylase in peripheral mononuclear blood cells temporarily decreased under infusion with paclitaxel equilibrating within 24 h to enzyme levels observed prior to therapy (Figure 1). In the next 48 hours, these levels decreased to thymidine phosphorylase levels significantly below pretherapeutic values resulting in a TP nadir on day 3 (p = 0.023). Only then, the expression of thymidine phosphorylase increased moderately reaching levels similar to the baseline levels observed in paclitaxel naive patients. The overall picture in week 6 was very similar to that observed in week 1 with a nadir of thymidine phosphorylase expression on day 3 after drug administration (Figure 2). Considering the flat concentration-time curve in patients, the calculation of the parameters time to maximum expression (t_max_) and maximum concentration in blood cells (c_max _at t_max_) could not be performed. Similarly, the slope of the trend analysis (observation period: 15 days) was not significantly different from zero indicating a stable enzyme expression in blood cells. The maximum expression level (steady-state) of thymidine phosphorylase in human peripheral blood cells after repeated administration of 80 mg paclitaxel/m^2 ^i.v. in week 6, i.e. after six doses of paclitaxel, was very similar to that observed in paclitaxel naive patients, but also to that after repeated administration of paclitaxel. This congruence is indicative for a stable phenotype of TP expression in peripheral mononuclear blood cells, which is subject to little variation for at least eight weeks in humans. Table 2 lists the arithmetic means ± standard deviation, 95% confidence intervals (CI), and outliers of every measurement.

**Table 2:** Results

Week	Measurement	Results (TP ng/ml) (mean +/- SD; 95% CI; outliers)	
1	1	299.24 ± 35.09; 95% CI 199.09 – 258.58;	283.42; 169.49
	2	182.97 ± 56.18; 95% CI 136.00 – 229.93;	240.39; 93.80
	3	246.77 ± 70.14; 95% CI 188.13 – 305.41;	350.41; 173.99
	4	155.20 ± 72.11; 95% CI 94.92 – 215.49;	242.42; 49.57
	5	170.66 ± 47.63; 95% CI 130.84 – 210.47;	232.71; 115.57
	6	187.55 ± 67.24; 95% CI 131.33 – 243.76;	295.48; 79.59
	7	200.79 ± 105.45; 95% CI 112.64 – 288.95;	369.96; 17.24
	8	196.36 ± 139.83; 95% CI 79.46 – 313.27;	431.39; 4.74
6	1	233.98 ± 52.29; 95% CI 190.26 – 277.70;	297.52; 125.95
	2	177.54 ± 76.06; 95% CI 113.95 – 241.13;	258.41; 50.44
	3	179.14 ± 76.68; 95% CI 115.03 – 243.24;	273.87; 43.42
	4	110.93 ± 66.80; 95% CI 55.08 – 166.77;	187.07; 20.76
	5	183.81 ± 52.89; 95% CI 139.59 – 228.02;	228.32; 60.86
	6	185.02 ± 70.87; 95% CI 125.77 – 244.27;	251.34; 43.74
	7	232.50 ± 126.58; 95% CI 126.67 – 338.32;	371.45; 6.62
	8	215.03 ± 90.78; 95% CI 139.13 – 290.92;	297.99; 7.56

## Discussion

In this trial, neither a short- nor a long-term induction of thymidine phosphorylase was observed in peripheral mononuclear blood cells under weekly therapy with paclitaxel. Considering the observation period of 7 weeks in total, thymidine phosphorylase was hardly regulated in human cells either under therapy or after termination of paclitaxel administration.

As a consequence, the assumed synergism between capecitabine and paclitaxel due to TP induction needs to be discussed covering aspects of haematotoxicity as well as therapeutic efficacy.

Capecitabine in general is regarded as cytotoxic agent with relatively low haematologic toxicity. In a small, yet randomized trial comparing capecitabine and paclitaxel in anthracyclin-pretreated patients, grade 3/4 neutropenia was observed in only 9% of patients on capecitabine as compared to 53% in the paclitaxel arm [17]. A more recent study evaluated the pharmacogenetics of capecitabine in advanced breast cancer patients. In the 105 patients included, grade 3/4 haematotoxicty was observed in 6% only [18]. The same 6% grade 3/4 haematotoxicity rate was reported in an observational study of capecitabine in anthracycline- and taxane-pretreated patients [19]. Taxanes, on the other hand, are known to be associated with major haematological side effects, which appear to be most pronounced with docetaxel. A large randomized trial comparing the two taxanes in metastatic breast cancer (both with a three weekly regimen) reported grade 3/4 neutropenia in 93.3% percent of patients treated with docetaxel, while only 54.5% of patients on paclitaxel experienced grade 3/4 neutropenia (p < 0.0001). The same holds true for the incidence of febrile neutropenia (14.9% in the docetaxel arm versus 1.8% in the paclitaxel arm [p < 0.001]) [20].

Based on the assumed synergistic effect of taxane plus capecitabine combinations attributed to the induction of TP in tumour tissue, a large randomized study comparing docetaxel as single agent with docetaxel plus capecitabine combination in the first line therapy of metastatic breast cancer was initiated [21]. Combination of these cytotoxic agents resulted in improved overall response rate and time to disease progression. Notably, addition of capecitabine did not add major haemotologic toxicity when compared to docetaxel monotherapy. A recent trial conducted by Blum et al evaluated the combination of weekly paclitaxel, which is known to yield better results as compared to the three weekly schedule, with capecitabine. Again, grade 3/4 neutropenia was experienced by 13% of patients only [10]. It may therefore be stated that capecitabine adds little toxicity to taxane therapy considering neutropenia. Our data may in part explain the underlying mechanism: While potentially TP activity is increased in tumour tissue by taxane exposure, such increase is obviously not the case in peripheral mononuclear cells. Therefore, we assume that no synergistic haematotoxicity is to be expected. Especially the temporary decrease of TP levels in blood cells clearly points at a favourable time profile for the combination of both compounds. Our results however are limited by two factors. First, although a total of 128 samples were collected, statistical power remains relatively low with only eight patients included. Secondly, the main haematological toxicity of paclitaxel is neutropenia, i.e. a lack of polymorphonuclear white blood cells. While the use of peripheral mononuclear blood cells is convenient for study reasons, it is therefore possibly not the ideal compartment to evaluate haematological toxicity in detail.

Results of this trial must also be discussed in the light of the assumed synergistic interaction of taxanes and capecitabine concerning tumour therapy. Considerable knowledge about TP induction has been obtained, yet data stem from preclinical models, even though the hypothesis tested in those investigations has already been transferred into clinical practice [6,8-10]. Concerning experimental data, a closer look at some of the published studies does not lead to unequivocal results. Sawada et al reported a clear induction if thymidine phosphorylase in the mouse model by paclitaxel. On the other hand, no major upregulation of TP in the cell culture was observed [4]. Importantly, in this trial an upregulation of human tumour necrosis factor α (TNFα) in the xenograft model was seen, which correlated strongly with increased TP levels. It is therefore assumed that paclitaxel induces TNFα in stromal cells of tumour tissue, which in turn holds responsible for the increase of TP levels. This might further explain, why no TP upregulation was observed in cell culture, and also our trial did not show an upregulation in peripheral mononuclear cells. Further, the kinetics of TP induction in tumour cells remain less then clear: There is an early observation of poor activity in mice when another taxane, docetaxel and capecitabine are administered simultaneously or when the taxane is administered after 14 days of continuous capecitabine treatment [7]. A similar observation has been reported in cell culture experiments combining paclitaxel and another prodrug of 5-fluorouracil, furtulon, in ovarian cancer *in vitro*, even after induction of thymidine phosphorylase [22]. Therefore, further investigation of taxane associated TP induction in human tumours is warranted.

## Conclusion

We conclude that taxane and capecitabine combinations are a valuable treatment option in advanced breast cancer with an acceptable toxicity profile. As shown in our trial, no increase of haematologic toxicity by TP induction in white blood cells needs to be expected. To our best knowledge, these are the first data about longitudinal expression of TP in humans under therapy with paclitaxel. Considering the lack of information about the modulation of TP in tumour tissue, it is not clear whether peripheral mononuclear cells may serve as valid surrogate parameter for TP levels in the malignant lesion. In addition, TP increase in tumours may depend on functional tumour – stroma interaction. Thus, trials evaluating this effect in humans are urgently warranted.

## Competing interests

The author(s) declare that they have no competing interests.

## Authors' contributions

RB participated in the study coordination, and drafted the manuscript, GS participated in the design of the study and revised the manuscript critically, BF and BR both carried out the assays, CW participated in the statistical analysis and helped in the collection of patient data, UP participated in the collection of patient data, CZ was important in the study coordination. RM contributed the idea for this trial, helped drafting the manuscript and performed the statistical analysis.

All authors have read and approved the final manuscript.

## Pre-publication history

The pre-publication history for this paper can be accessed here:


